# Extracellular matrix stiffness mediates radiosensitivity in a 3D nasopharyngeal carcinoma model

**DOI:** 10.1186/s12935-022-02787-5

**Published:** 2022-11-19

**Authors:** Yanhua Fang, Shanshan Liang, Jianong Gao, Zhe Wang, Cheng Li, Ruoyu Wang, Weiting Yu

**Affiliations:** 1 The Key Laboratory of biomarker high throughput screening and target translation of breast and gastrointestinal tumor, Affiliated Zhongshan Hospital of Dalian University, No.6 Jiefang Street, Zhongshan District, Dalian, 116001 Liaoning China; 2 Oncology Department, Affiliated Zhongshan Hospital of Dalian University, No.6 Jiefang Street, Zhongshan District, Dalian, 116001 Liaoning China; 3 Outpatient Department, General Hospital of Northern Theater Command, No.83 Culture Road, Shenhe District, Shengyang, 110015 Liaoning China; 4 Affiliated Zhujiang Hospistal of Southern Medical University, Zhongshan Hospital of Dalian University, 253 Industrial Avenue, 510280 Guangzhou, People’s Republic of China

**Keywords:** Nasopharyngeal carcinoma, Radioresistance, Extracellular matrix, Stiffness, 3D culture model

## Abstract

**Purpose:**

Radiotherapy is one of the essential treatment modalities for nasopharyngeal carcinoma (NPC), however, radioresistance still poses challenges. Three-dimensional (3D) tumor culture models mimic the in vivo growth conditions of cells more accurately than 2D models. This study is to compare the tumor biological behaviors of NPC cells in 2D, On-Surface 3D and Embedded 3D systems, and to investigate the correlation between radioresistance and extracellular matrix (ECM) stiffness.

**Methods:**

The morphology and radioresistance of the human NPC cell line CNE-1 were observed in 2D and 3D systems. The CCK-8 assay, wounding healing assays, flow cytometry, soft agar assays, and western blot analysis were used to evaluate differences in biological behaviors such as proliferation, migration, cell cycle distribution, and stem cell activity. Different ECM stiffness systems were established by co-blending collagen and alginate in varying proportions. ECM stiffness was evaluated by compressive elastic moduli measurement and colony formation assay was used to assess radioresistance of NPC cells in systems with different ECM stiffness after irradiation.

**Results:**

Compared to 2D models, the morphology of NPC cells in 3D culture microenvironments has more in common with in vivo tumor cells and 3D cultured NPC cells exhibit stronger radioresistance. Integrin β1 but not the epithelial-to-mesenchymal transition pathway in 3D models boost migration ability. Cell proliferation was enhanced, the proportion of tumor stem cells was increased, and G1/S phase arrest occurred in 3D models. NPC cells cultured in softer ECM systems (with low alginate proportions) exhibit striking resistance to ionizing radiation.

**Conclusion:**

The tumor biological behaviors of NPC cells in 3D groups were obviously different from that of 2D. Radioresistance of NPC cells increased with the stiffness of ECM decreasing.

**Supplementary Information:**

The online version contains supplementary material available at 10.1186/s12935-022-02787-5.

## Introduction

Nasopharyngeal carcinoma (NPC) is a regional tumor with more than 70% of new cases reported in East and Southeast Asia [1]. The main method of controlling NPC locally is radiotherapy. However, radioresistance, which leads to recurrence and metastasis, has been the main cause of death [2–4]. Two-dimensional (2D) culture-based radioresistance models are still widely employed in radiobiological research today. Radioresistance models are typically formed using conventional fractionated radiotherapy (CFRT), which delivers multiple doses of 2–6 Gy over several rounds, reaching a total dose of 60–80 Gy [5, 6]. 2D radioresistance models are monolayer systems that lack the tumor microenvironment and cell–cell contact, which are key factors in radiobiology. Additionally, low-dose fractionated irradiation also leads to long-term instability of the genome [7]. In 3D culture models, cells are given access to synthetic or natural substances that resemble the extracellular matrix (ECM), where they can develop in close proximity to other cells and at gradient concentrations of cytokines needed for particular biological processes. The response of chemotherapy and radiotherapy has recently been predicted using 3D cultures, primary cultures, and xenograft tumors [8–10]. 3D culture models showed more advantageous than 2D for drug testing and efficacy prediction [11, 12]. Therefore, it is necessary to establish a 3D tumor culture model for further research into the radiobiology of NPC in order to investigate the mechanisms causing radioresistance.

It is commonly acknowledged that the tumor microenvironment, for instance, the ECM, plays significant roles in preserving physiological processes, particularly in the development of cancer [13, 14]. ECM stiffness is a crucial physical characteristic. Stiffness is converted to mechanical force signals, which are sensed by cells and lead to downstream epithelial-to-mesenchymal transition (EMT) signaling, which plays a role in tumor invasion and metastasis [15]. The ECM is altered during tumor progression as a result of ECM deposition by matrix metalloproteinases or mechanical stress resulting from tumor growth, whereas the interstitial matrix is predominantly composed of substances such as collagens I and III, fibronectin (FN), and elastin. To our knowledge, the ECM composition in NPC has not been investigated in detail. Differences in ECM stiffness between tumor and normal tissues have been reported. An increase in collagen deposition, which enhances tissue stiffness and is related with tumor growth and metastasis, is the most common ECM modification associated with tumor formation [16]. Owing to an increase in FN cross-linking, the ECM of tumors, which is predominantly made of fibrous tissue, is stiffer than the ECM of normal tissue.

Few direct studies have been conducted on the stiffness and radioresistance of tumor cells. Triple-negative breast cancer cells interacting with soft ECM were confirmed to exhibit striking resistance to ionizing radiation [17]; however, increasing amounts of indirect evidence are emerging, such as the finding that the stiffness of the matrix affects the expression of tumor stem cell markers in breast and colon cancer [18]; and that extracellular mechanical changes regulate DNA double-strand break (DSB) repair processes [19]. These findings indicate that ECM stiffness functions in tumor resistance to therapy and that interference with matrix stiffness may affect drug resistance. Therefore, tumor ECM stiffness is typically a target for modeling the tumor microenvironment and sensitizing therapies[16]. To study the effects of ECM stiffness on tumor physiological activity and the underlying mechanisms in vitro, cancer cells are usually grown in 3D culture matrices with collagen, sodium hyaluronate, or other scaffold materials that mimic the ECM [20]. An interpenetrating polymer network (IPN) is a special type of polymer structure created by the networked interaction of two or more polymers [21]. Collagen/alginate IPN hydrogels are widely used as scaffolds to study the biological behavior of cells.

Here we established two kinds of 3D culture models, on-surface and embedded, of the human NPC cell line CNE-1, to compare the radioresistance of 3D and 2D cultured tumor cells. We examined the biological differences and associated markers to determine why the tumor cells grown in 3D culture are more radioresistant. Finally, we co-blended collagen and alginate to create IPN hydrogels with different stiffness and demonstrated that the radioresistance of on-surface 3D tumor cells was affected by the stiffness of the ECM. This could offer a new perspective for 3D culture tumor models in radioresistance and radiosensitization research.

## Materials and methods

### Preparation of collagen

Rat tail tendons were extracted using hemostatic forceps, immersed in 75% ethanol for 5 min, and placed in saline solution. After they were sliced, 0.1 wt% acetic acid solution was added to the tail tendon fragments and they were maintained at 4 °C for 7 days. After centrifugation of the combined liquid for 15 min at 5000 rpm, the supernatant was removed. The rat tail collagen solution was validated using SDS-PAGE, tested for sterility, freeze-dried, and maintained at − 20 °C (extended storage at − 80 °C). Before use, 6 mg/mL and 2 mg/mL collagen solutions were created by dissolving the lyophilized rat tail collagen in 0.1 wt% acetic acid.

### Cell culture

Human nasopharyngeal carcinoma cell line CNE-1 (Presented by Sun Yat-sen University, Guangzhou, China, passage 20) was used as a representative cancer cell. Cells were cultured in RPMI-1640 (Gibco) supplemented with 10% fetal bovine serum (Gibco) and 1% penicillin–streptomycin (Hyclone, Logan, UT, USA) at 37 °C with 5% CO_2_. Cells were passaged with 0.25% trypsin/EDTA (Hyclone). *Mycoplasma* infection was routinely tested by PCR (CycleavePCR® Mycoplasma Detection Kit, TaKaRa, Cat. No. CY232).

### CCK-8 proliferation assay

Standard curve: First, 1000, 2000, 4000, 8000, 16,000, 32,000, 64,000, and 128,000 CNE-1 cells were plated in a 96-well plate (*n* = 6), followed by incubation for 4 h. Next, 10 µL of CCK-8 reagent was added to the wells, and the cells were incubated for another 4 h at 37 °C. At 2, 3, and 4 h after applying the CCK-8 reagent, the optical density (OD) was measured using a microplate reader (BIO-RAD, CA, USA) to create the standard curve.

Proliferation curve: First, 2000 cells/well in the 2D group, 10,000 cells/well in the on-surface 3D group, and 20,000 cells/well in the embedded 3D group were inoculated in 96-well plates (*n* = 6) based on the proliferation differences between the cells in each group. Each well’s OD value from the previous six days was read, and the standard curve was regressed to get the appropriate number of cells for drawing the growth curve.

### Construction of the 3D cell culture system

**Fig. 1:**
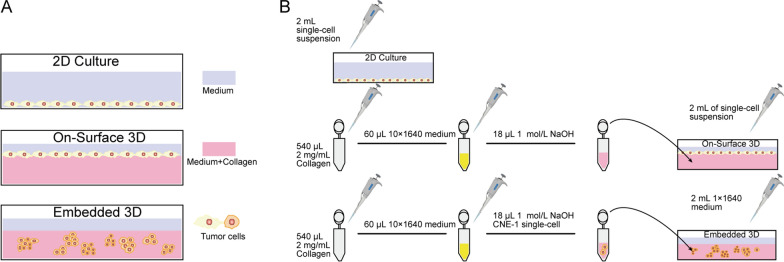
Schematic diagram of cell culture systems **A** Diagram of 2D and 3D cultivation models. **B** Schematic diagram of the components of a typical culture system

Figure 1 A shows how the 2D and 3D culture models were established.

As shown in Fig. 1B, for the 2D group, 2 mL single-cell suspension with 4 × 10^4^ cells/well was added to a 6-well plate. For the on-surface 3D group, 540 µL of 2 mg/mL collagen solution and 60 µL of 10 × 1640 medium were added into a 1.5-mL EP tube. Next, 18 µL of 1 M NaOH solution was added to adjust the pH value to 7–7.4. The mixed solution was then pipetted into the 6-well plates. After 2 h at 37 °C, collagen was coagulated. Next, 2 mL of single-cell suspension with 6 × 10^4^ cells/well was added to the plates. For the embedded 3D group, a concentrated cell suspension of 2 × 10^5^ cells was added to the alkaline-adjusted collagen–medium mixture. After mixing, the solution was pipetted into 6-well plates, where it coagulated for 2 h. After 2 mL of 1 × 1640 medium was added, cells were grown at 37 °C with 5% CO_2_ for 3 days.

### Construction of the hydrogel adjusting stiffness culture system

In this section, the 2D culture group served as the control group. First, 2 mL of single-cell suspension of 4 × 10^4^ cells/well was added to the 6-well plates. The following materials were used to produce collagen and alginate hydrogel with varying stiffness: stock solutions of 6 mg/mL collagen, 60 mg/mL sodium alginate, 0.9% NaCl, and 10 × 1640 medium. To achieve the necessary final concentration, 180 µL collagen solution, varied volumes of sodium alginate, and matched volumes of 0.9% NaCl or 0.1% acetic acid were utilized, followed by the addition of 18 µL of 0.1 M NaOH to adjust the pH of each solution to 7–7.4. Each solution was pipetted into the 6-well plates in order to coagulate. Then, 3 mL of 0.1 M CaCl_2_ solution was quickly injected into wells containing sodium alginate, which were allowed to stand for 10 min, replacing sodium alginate with calcium alginate. Next, 2 mL of cell suspension containing 2 × 10^5^, 1.6 × 10^5^, 1.2 × 10^5^, 8 × 10^4^, or 6 × 10^4^ cells was added to each 3D well, followed by incubation at 37 °C for 3 days. Detailed information is provided in Table 1.

**Table 1:** List of compound volume in different groups

Group	Modulus Kpa	Collagen	Sodium alginate	10 × 1640 medium	0.9% NaCl	0.1% acetic acid	0.1 mol/L NaOH	0.1 mol/L CaCl_2_	Cells
2 mg/mL collagen+ 40 mg/mL Alg	208.2 Kpa	180 µL	360 µL	60 µL	-	-	18 µL	3 mL	2 × 10^5^
2 mg/mL collagen+ 20 mg/mL Alg	94.99 Kpa	180 µL	180 µL	60 µL	180 µL	-	18 µL	3 mL	1.6 × 10^5^
2 mg/mL collagen+ 10 mg/mL Alg	50.74 Kpa	180 µL	90 µL	60 µL	270 µL	-	18 µL	3 mL	1.2 × 10^5^
2 mg/mL collagen+ 5 mg/mL Alg	10.6 Kpa	180 µL	45 µL	60 µL	315 µL	-	18 µL	3 mL	8 × 10^4^
2 mg/mL collagen	-	180 µL	-	60 µL	-	360 µL	18 µL	-	6 × 10^4^
2D	-	-	-	-	-	-	-	-	4 × 10^4^

### Cytoskeleton protein assay

The culture medium of each group was removed. Cells were fixed with 4% paraformaldehyde solution for 15 min, followed by permeabilization with 0.1% Triton TM X-100 (Solarbio, Beijing, China) for 15 min. Next, 1% BSA solution was added followed by incubation for 30 min to block the cells, and 1× FITC-phalloidin (Invitrogen, California, USA) diluted in DMSO was added followed by incubation for 1 h. Finally, 2.5 µg/mL DAPI (Solarbio) was added dropwise into the wells. Cells were observed under a laser scanning confocal microscope (Leica).

### Cell irradiation

The medical linear accelerator (Varian, MA, USA) was used for X-ray irradiation. The irradiation conditions were: 6 Mv X-rays, 600 cGy/min dose rate, source skin distance 100 cm, DT = 0, 2, 4, 6, 8 Gy, irradiation field 10 cm × 10 cm.

### Colony formation experiment

The culture medium was removed after different dosages of X-ray irradiation (0, 2, 4, 6, and 8 Gy) were administered. The collagen medium from the 3D groups was transferred into a centrifuge tube, 2 mL of 0.3 mg/mL collagenase was added, and the mixture was incubated at 37 °C for 10 min. The digestion was terminated with serum-containing medium, samples were centrifuged at 800 rpm for 5 min, and the supernatant was discarded. The sediment was incubated with 300 µL 0.25% trypsin at 37 °C for 3 min. The digestion was again halted with serum-containing medium, and then the cells were centrifuged and inoculated into 6-well plates at a concentration of 300 cells per well (*n* = 3). When 0 Gy in the 2D group produced single-cell colonies containing 50 cells, the colonies were washed twice with PBS, fixed with 4% paraformaldehyde for 15 min, dyed with 0.1% crystal violet solution for 30 min at room temperature, and then photographed. Colonies with more than 50 cells were manually counted. ImageJ was used to determine the colony diameter. Under each radiation dose, the survival fraction (SF) was calculated as follows: SF = the number of colonies formed after irradiation/(number of seeded cells × colonies formed of non − irradiated cells) × 100%.

### Wounding healing assay

CNE-1 cells grown for 3 days in three different systems were digested and seeded in 6-well plates at a density of 3.8 × 10^5^ cells/well, followed by incubation overnight (the fusion rate reached 100%). The supernatant was filtered and the monolayer was wounded by sterile 200-µL pipette tips. The monolayer was washed with serum-free medium and photographed (0 h). The cells were cultured with serum-free medium for 3, 6, 9, 12, and 24 h to test the migratory capabilities. The wound closure ratio was calculated as follows: wound closure ratio = (wound width at 0 h − wound width at *X* h)/wound width at 0 h.

### Transwell assay

CNE-1 cells cultured for 3 days in the three systems were digested and centrifuged at 1000 revolutions per minute (rpm) for 5 min, then 1 × 10^5^ cells in 100 µL serum-free RPMI 1640 medium were added to the upper layer of Transwell chamber (8.0 μm, Corning, NY, USA). 700 µL RPMI 1640 culture medium with 5% FBS was added to the lower chamber and were incubated at 37 °C for 12 h. After crystal violet staining, five fields were randomly selected and photographed (×100). The number of migratory cells was counted.

### Flow cytometry

The cells of each group were harvested, and 3 mL of precooled 70% ethanol was added while swirling. After fixation at − 20 °C overnight, samples were centrifuged to remove the ethanol and cells were washed with PBS. The cells were suspended in ice-cold PBS and counted. Then, to every 1 × 10^6^ cells in suspension, 500 µL PI/RNase (Roche, Shanghai, China) was added, followed by incubation in the dark at room temperature for 15 min. The cell cycle distribution was analyzed by a BD flow cytometer (Franklin Lakes, NJ, USA).

### Stiffness measurements

Compressive elastic moduli of hydrogels were measured. Briefly, hydrogel samples were prepared as cylindrical gels with a diameter of 3 mm and a thickness of 1 mm and gels were compressed with a MicroSquisher in a water bath. The compressive modulus was determined from the slope of the linear region on the stress–strain curve (*n* = 5 per condition).

### Soft agar assay

A total of 5000 cells were resuspended in 1.5 mL of the top layer of agarose media (0.3% agarose) and plated on top of the bottom agarose layer (0.5% agarose) in 6-well plates. Next, 1 mL medium was added to the top layer of agar. Colonies formed in 10–14 days after the top medium was changed every 3 days. The colonies were stained for 2 h with a 5 mg/mL MTT solution and photographed.

### Western blot

Total protein was extracted from cell samples using RIPA Lysis Buffer (Sigma, St. Louis, MO, USA) mixed with phosphatase inhibitor and protease inhibitor (Roch). The concentration of protein was quantified using Bradford Protein Assay Kit (Sangon Biotech). After mixed with 5 × loading buffer, denaturation at 100 °C for 8 min, protein samples were loaded, subjected to 10% sodium dodecyl sulfate (SDS)-polyacrylamide gel electrophoresis (PAGE) and transferred to 0.2 μm polyvinylidene fluoride (PVDF) membranes (Millipore, Massachusetts, USA). After being blocked by 5% skim milk for 1 h, these membranes were incubated with primary antibody at 4 °C overnight. Antibody information and dilution ratio are as follows: E-cad (CST, Massachusetts, USA, Cat. No. 3195, 1:1000), Vimentin (CST, Cat. No. 5741, 1:1000), N-cadherin (CST, Cat. No. 4061, 1:1000), Slug (CST, Cat. No. 9585, 1:1000), ZEB-1(CST, Cat. No. 3396, 1:1000), CD44(CST, Cat. No. 3570, 1:1000), SOX-2 (CST, Cat. No. 3579,1:1000), Integrin β1 (Sigma, Cat. No. SAB4300655, 1:500), CDk4 (Proteintech, Illinois, USA, Cat. No. 11026-1-AP, 1:2000), Cyclin D1 (CST, Cat. No. 2978, 1:1000), Cyclin E1(Abcam, Cambridge, MA, Cat. No. ab33911, 1:1000), GAPDH (CST, Cat. No. 5174 S, 1:8000). Then, the PVDF membranes were washed in TBST buffer and incubated with IgG-HRP secondary antibody (CST, Cat. No. 7076/7074, 1:8000) for 1 h at room temperature. After being washed in TBST buffer again, proteins were visualized with electrochemiluminescence substrate (Advansta, California, USA) and the strips were analyzed and photographed by Tanon-5200 automatic chemiluminescence imaging analysis system (Tanon, Shanghai, China).

### Statistical analysis

Data were analyzed using GraphPad Prism 7 (Version X; California, USA). Statistical analysis was performed by Student’s t test. *P* value < 0.05 was considered as statistically significant. The number of biological replicate experiments is denoted by *n*.

## Results

### Cell morphology and cytoskeleton distribution in 3D and 2D culture

**Fig. 2:**
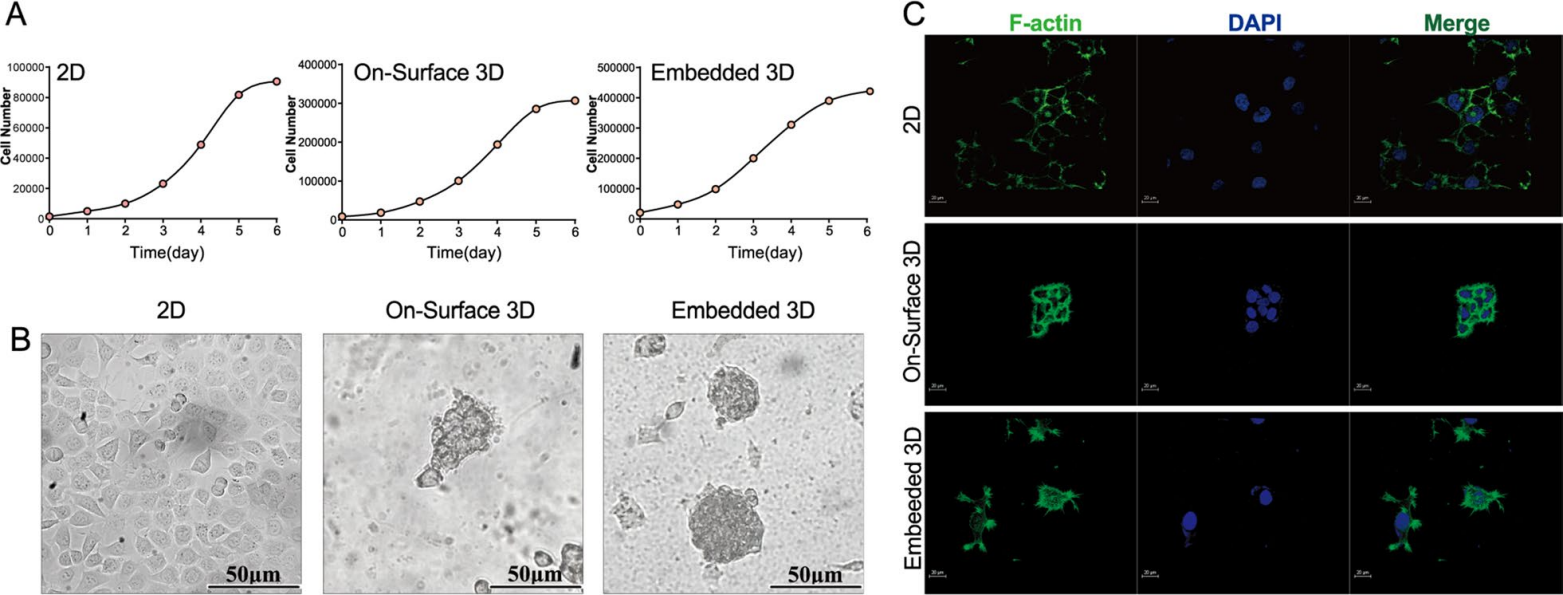
Cell morphology and cytoskeleton distribution in 3D and 2D systems were different **A** Growth curves of CNE-1 cells in 2D, On-Surface 3D, and Embedded 3D group (*n* = 6). After 3 days of inoculation, cells were in the logarithmic phase of growth. **B** The growth morphology of CNE-1 cells cultured in three systems were significantly different (scale bar 50 μm). **C** Representative immunofluorescent images of skeleton protein distribution (scale bar 20 μm)

Two kinds of 3D (On-Surface 3D and Embedded 3D) and 2D culture systems of human NPC line CNE-1 were constructed as in Fig. 1. Due to different proliferation abilities, the initial cell number of cells in each group was 2000 for 2D group, 10,000 for On-Surface 3D and 20,000 for Embedded 3D, respectively. Cells were in the logarithmic growth phase on the third day so that subsequent tests were all carried out after three day’s “educated” in each system (Fig. 2 A). CNE-1 cells cultured in 2D and 3D exhibited considerably distinct morphologies. 2D cultured cells developed in a monolayer that was fully stretched and flat. The on-surface 3D cells grew in clusters and adhered to the bottom matrix without being fully stretched. The embedded culture created a collagen-encapsulated contact situation, causing the cells to exhibit a round shape similar to that of in vivo tumor cells (Fig. 2B). The distribution of the cytoskeleton protein F-actin was observed under a confocal microscope. F-actin of 2D cells was evenly distributed in the cytoplasm by a scatter-like manner, which played a role in supporting the spreading of cells. F-actin of cells in 3D culture had a smaller spreading area, thicker and stronger fluorescent staining pattern than 2D cultured cells (Fig. 2 C).

### 3D model “educated” NPC cells showed enhancement of radioresistance

**Fig. 3:**
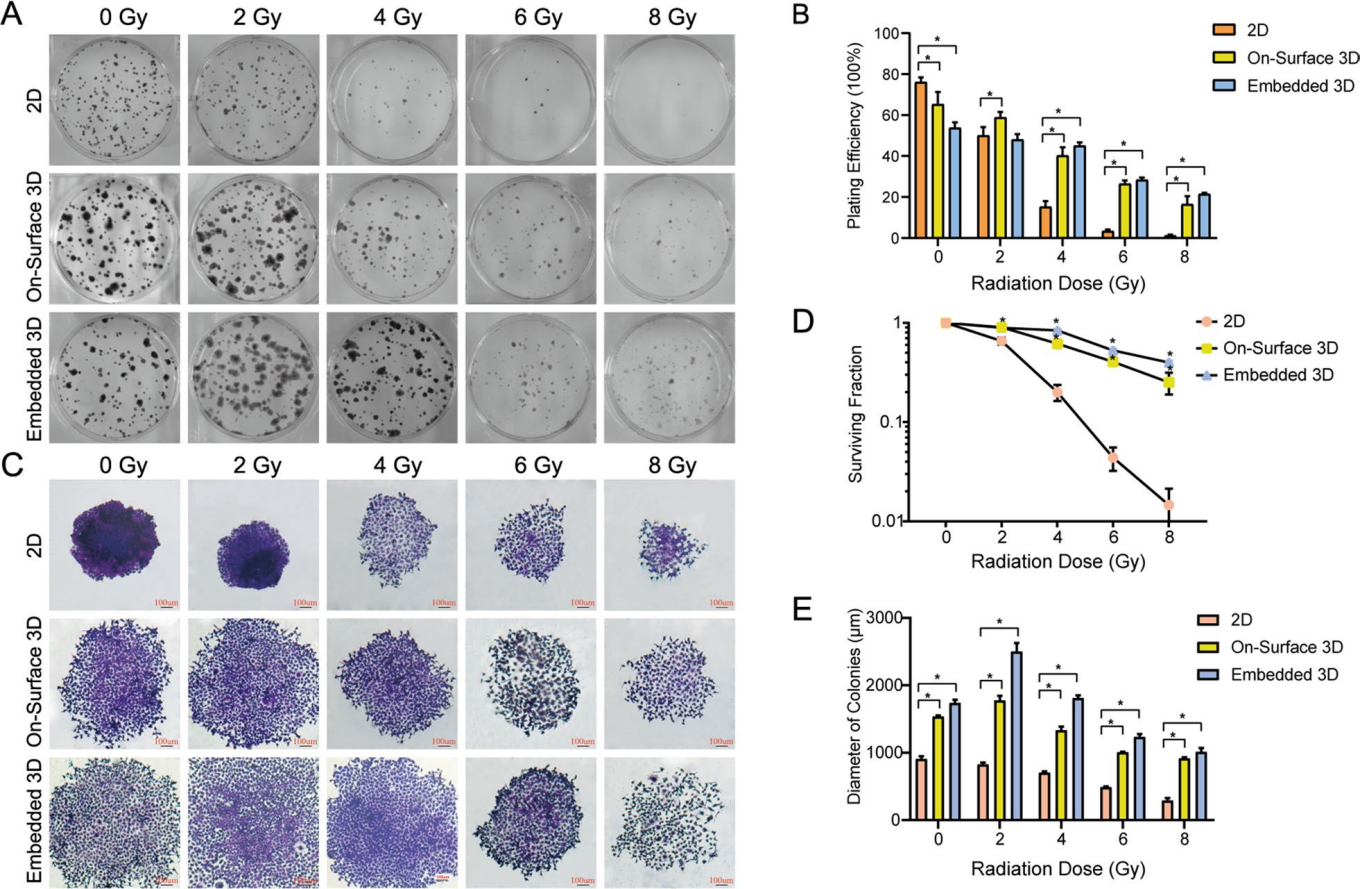
Cell radioresistance enhanced in 3D culture system **A** The colonies formed by CNE-1 cells cultured in 2D, On-Surface 3D, and Embedded 3D group after different doses of X-ray irradiation. **B** The plating efficiency and **D** surviving fraction of CNE-1 cells cultured in the three culture systems after receiving different doses of X-ray irradiation (*n* = 3, *P < 0.05). **C, E** Comparison of the colony diameters under different X-ray doses in the three systems (*P < 0.05)

After “educated” in three systems, CNE-1 cells were collected and irradiated by different doses of X-rays (2,4,6,8 Gy). The diluted solution of single cells was then used to form colonies. After 7 days, cells were fixed and dyed and colonies were counted (Fig. 3 A and C). As the irradiation dose increased, the plating efficiency of each group showed a decreasing trend. At 0 Gy, the plating efficiency of the two 3D groups (64.2% and 54%) was significantly lower than that of the 2D group (75%); however as the irradiation dose increased, the plating efficiency of 3D groups were higher than 2D (Fig. 3B). The survival fraction (SF) of two 3D groups presented higher than 2D group under each irradiation dose. At 8 Gy, the SF were 0.252 and 0.400 for On-Surface 3D and Embedded 3D group respectively, compared to 0.015 for 2D group (Fig. 3D). The 2D group’s mean colony diameter dropped with increasing irradiation dose and reached a maximum of 908 μm at 0 Gy. While, the mean colony diameters of On-Surface 3D and Embedded 3D group showed a tendency of first growing and then declining, with the maximum diameters occurring at 2 Gy, 1775 and 2498 μm respectively (Fig. 3E). These results revealed that 3D culture conditions improved the irradiation resistance of NPC cells.

### 3D model “educated” NPC cells represented enhancement of migration and adhesion

**Fig. 4:**
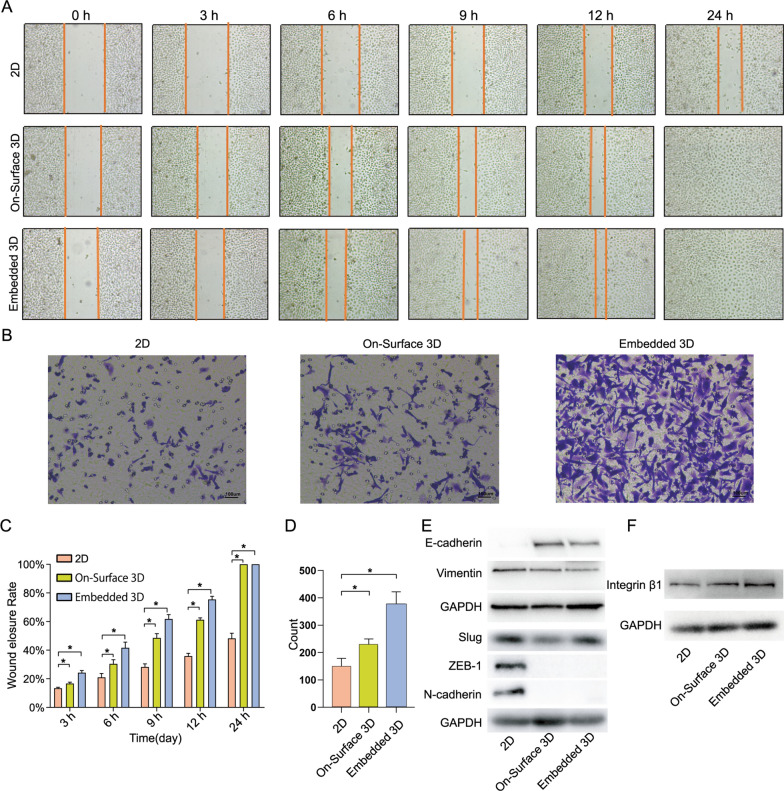
Cell migration and adhesion enhanced in 3D culture system **A** The migration of CNE-1 cells cultured in the three systems at 0, 3, 6, 9,12 and 24 h after scratch was significantly different (*n* = 3). **B** Migration capability of CNE-1 cells in each group detected by transwell assay. **C** The wound elosure rate in three groups at each time point was calculated by Image J software (*n* = 3, *P < 0.05). The cell migration rate of cells in the 3D groups were significantly faster than that in 2D. **D** The number of migratory cells in each group (*n* = 3, *P < 0.05). **E**, **F** Western blot analysis of E-cadherin, Vimentin, N-cadherin, Slug, ZEB-1 and Integrin β1 expression in cells of each group

After “educated”, CNE-1 cells were collected and seeded in six-well plates. The wound closure ratio in two 3D groups was significantly higher than 2D. At 24-hour, the wound closure ratio of both 3D groups was 100%, indicating that the cells had completely covered the scratch. Whereas the wound closure rate in the 2D group was only approximately 50% (Fig. 4 A, Fig. 4 C). The transwell assay was also used to determine the cells’ migration capability. Cells were seeded in the upper compartment, and after 12 h, cells were stained and counted. The number of migratory cells in the two 3D groups, 226 and 379, was significantly greater than in 2D, which was 151 (Fig. 4B and D). To investigate the migration-regulating mechanism of 3D cultured cells, the expression levels of the EMT-related markers E-cadherin, Vimentin, N-cadherin, Slug, and ZEB-1 were examined. The expression of E-cadherin in the two 3D groups was higher than that in the 2D group. In contrast, the expression of Vimentin, N-cadherin, Slug, and ZEB-1 almost had the opposite trend (Fig. 4E). Contrary to the results of the wound healing experiment and the general understanding of the tumor EMT pathway in 2D conditions, the expression of these five markers in 3D cultured tumor cells suggests that the EMT pathway may not be the primary mechanism regulating the migration of these cells. Therefore, we detected the expression of Integrin β1 (Fig. 4 F) and the levels of Integrin β1 in the two 3D groups were higher than in the 2D group.

### Enhancement of proliferation and G1/S phase arrest occurred in 3D “educated” model

**Fig. 5:**
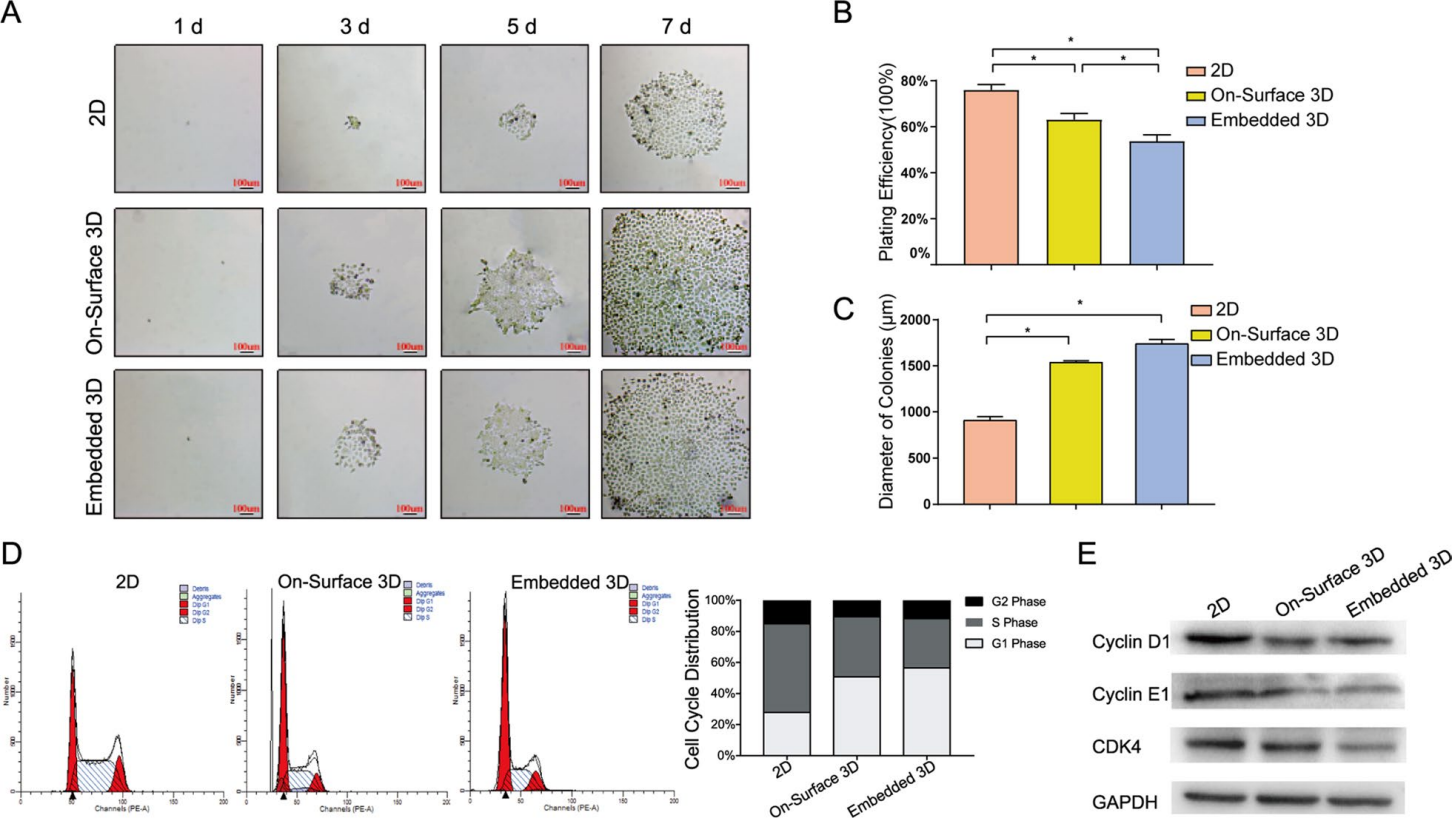
Cell proliferation was enhanced and G1 phase arrest occurred in 3D culture system **A** Cell colony formation in each group on day 1, 3, 5 and 7 (scale bar 100 μm). **B**, **C** Comparison of plating efficiency and colony diameter of CNE-1 cells cultured in three systems (*n* = 3, *P < 0.05). **D** The flow cytometry histogram of CNE-1 cells. The cell cycle distribution of CNE-1 cells in 3D groups arrested at G1 phase. **E** Western blot analysis of Cyclin E1, Cyclin D1 and CDk4 in three groups

After “educated”, cells were collected for colony formation and observed the colonies on the first, third, fifth, and seventh days. From the third day, it was obvious that the colony area of two 3D groups was greater, with the difference becoming more evident from the 5th to 7th day (Fig. 5 A). The plating efficiency of the embedded 3D group (53.6%) was lower than that of the on-surface 3D group (63.1%). The 2D group had the highest planting efficiency at 76% (Fig. 5B). While, the colony diameters showed precisely the opposite trend, 2D was significantly less than On-Surface/Embedded 3D groups (Fig. 5 C), indicating 3D culture may endow NPC cells with lower proliferation ability.

Flow cytometry was utilized to study the distribution of the cell cycle. In the two 3D groups, the proportion of cells in the G2 and S phases was lower, while the proportion of cells in the G1 phase was higher (Fig. 5D), indicating that the cell cycle of 3D cultured cells became more static. The expression levels of cyclins and CDKs associated with checkpoints were examined. The expression levels of cyclin D1 and CDK4, which govern the transition of cells from the G1 to the S phase, were reduced in the two 3D groups. The expression of cyclin E1, which plays a crucial function in the onset of the S phase, was decreased as well (Fig. 5E). These factors finally resulted in G1/S phase arrest in 3D culture groups.

### 3D models increase NPC cells stemness

**Fig. 6:**
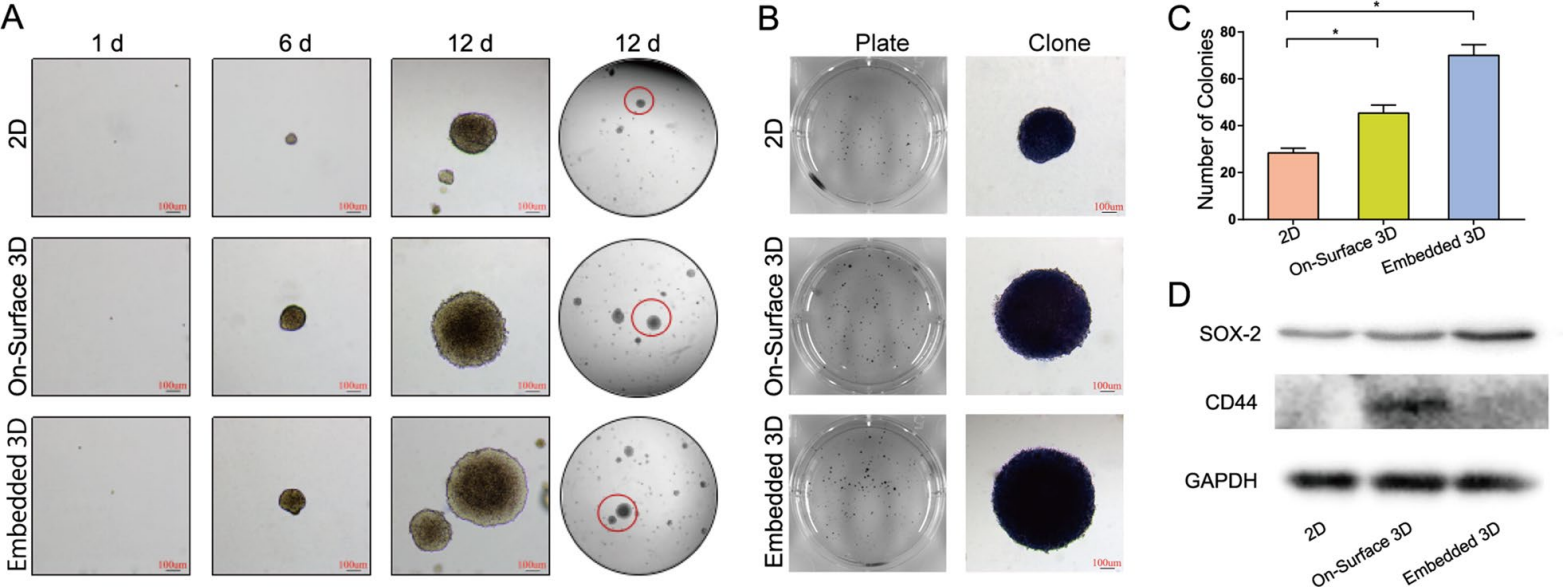
Proportion of tumor stem cells increased in 3D culture system **A** The formation of soft agar colonies was significantly different in each group on the 1st, 6th, and 12th day (scale bar 100 μm). **B** After fixation and staining, the well was photographed and the colonies were observed under the microscope (scale bar 100 μm). **C** The number of soft agar colonies in each group (*n* = 3, *P < 0.05). The number of tumor stem cells in the two 3D groups was significantly increased. **D** Western Blot analysis of SOX-2 and CD44 expression in each group. The expression of SOX-2 and CD44 were enhanced compared with 2D group

Cancer stem cells (CSCs) are a tiny population of tumor cells that share stem cell characteristics including self-renewal, proliferation, differentiation, and tumorigenicity. They are the primary determinants of tumor genesis, development, invasion, and metastasis. As a result, we utilized soft agar assay and western blot to determine the number and expression of cancer stem cell markers. 5000 cells were added to each well in the 6-well plate. The formation of cell colonies in each group was observed under microscope on days 1, 6 and 12 (Fig. 6 A). After staining, the well was photographed and the colonies were examined under a microscope (Fig. 6B). The number of colonies in 3D groups (45 and 70 respectively) were significantly increased compared with 2D group, whose number was 28 (Fig. 6 C). The expression levels of SOX-2 and CD44 were much higher in the two 3D groups (Fig. 6D). Theses results indicate that the proportion of CSCs in the 3D groups were significantly higher than that in the 2D group.

### ECM stiffness mediates radioresistance in 3D “educated” model

**Fig. 7:**
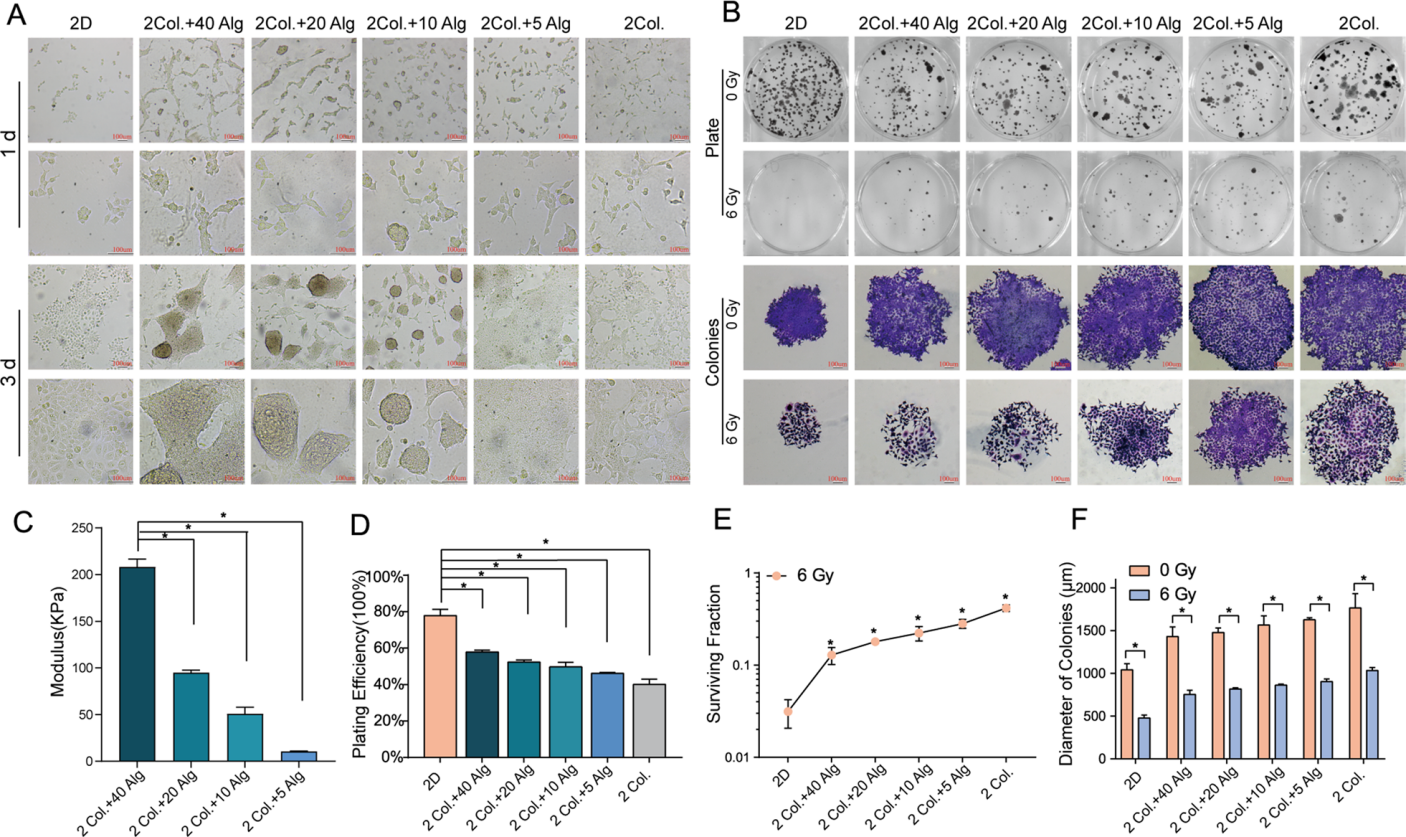
Radiation resistance decreased as the stiffness of On-Surface 3D matrix decreased **A** Effect of different surface stiffness of hydrogel on the morphology of CNE-1 cells on 1st, 3rd day (scale bar 100 μm). The lower concentration of sodium alginate, the more adherence, extension, connectivity of cells represented. **B** The formation of colonies after irradiated with 0 and 6 Gy X-rays cultured on different matrix stiffness (scale bar 100 μm). **C** Stiffnesses of hydrogel On-Surface 3D culture system consisting of permanent concentration of collagen and different concentrations sodium alginate. With the concentration of sodium alginate decreasing, shear stiffness declined. **D** The proliferation of CNE-1 cells on different stiffness substrates reflected by planting efficiency of colony formation assay. As the proportion of sodium alginate decreased (i.e., the stiffness decreased), the planting efficiency gradually descend. **E** The surviving fraction (6 Gy, *n* = 3, *P < 0.05) increased with the matrix stiffness declining. **F** Diameter of colonies increased with the matrix stiffness declining (*P < 0.05)

According to prior experiment on radioresistance (Fig. 3), On-surface 3D culture tumor cells can acquire a level of radioresistance similar to that of embedded 3D culture system. We created a hydrogel On-Surface 3D culturing system with varying stiffness by combining collagen with varying amounts of alginate. To determine the range of achievable stiffness, the shear modulus of hydrogels with alginate concentrations ranging from 40 to 5 mg/mL in the presence of permanent 2 mg/mL collagen was evaluated. As the alginate concentration decreased, ECM stiffness decreased (Fig. 7 C). The morphology of CNE-1 cells considerably changed after 3 days of culture on the surfaces of a hydrogel with tunable rigidity. The lower the alginate content, the softer the hydrogel surface and the more adherence, extension, and connectivity of cells were observed (Fig. 7A). After successfully establishing the hydrogel culture system with varying stiffness, the colony formation assay was performed to determine the proliferation of CNE-1 cells on substrates with different stiffness. The colonies were examined under a microscope on the first, third, fifth, and seventh days (Additional file 1: Fig. S1A). As the concentration of alginate decreased (i.e., lower 3D surface stiffness), the plating efficiency decreased gradually (Fig. 7D). However, the colony diameter followed an opposite pattern (Additional file 1: Fig. S1B). After X-ray irradiated at 0 and 6 Gy, CNE-1 cells cultured on matrices with different stiffness were digested, seeded in a 6-well plate (300 cells/well), and incubated for 7 days for the colony formation assay. As the concentration of alginate was decreased (lower stiffness), the colony diameter and SF increased gradually (Fig. 7B, E, and F). This suggests that the radioresistance of CNE-1 cells increased as the hydrogel stiffness decreased.

## Discussion

The tumor microenvironment contributes to tumor development, progression, and resistance to therapy. A 3D culture model is an ideal in vitro approach for studying the tumor microenvironment, which includes the ECM, cell–cell adhesion, and hypoxia. Currently, tumor spheroids, scaffold-based approaches, bioreactors, microfluidic devices, and organoids are utilized to retain and develop tumor cells in 3D culture [22, 23]. Our 3D culture models belong to scaffold-based hydrogel technologies, which offer the advantages of low cost and a realistic in vivo environment. Additionally, this tunable structural configuration facilitates control of the spatial distribution of cells and the ECM [24]. We developed two types of 3D culture systems and discovered that the growth of Embedded 3D CNE-1 cells more closely resembled the in vivo condition and had the highest radioresistance. This might be considered evidence that 3D cultures replicate the tumor microenvironment, including the ECM, cell–cell interactions, and signal transduction pathways, which are highly connected with cell behavior and resistance to chemotherapy or radiotherapy [25]. It is interesting to note that the cells from the 2D and on-surface 3D groups were both implanted onto the plate, the only distinction being that the On-surface 3D cultured cells can partially grow into collagen. However, On-surface 3D cultured cells can gain radioresistance qualities similar to those of the embedded 3D group. In addition, this phenomenon was also observed in breast cancer cell line MCF-7 (data not published). This revealed that the degree of cell adhesion (matrix stiffness) may affect the cells’ radioresistance in 3D culture systems. Before validating this assumption, we first determined biological properties of the cells, including proliferation, migration, cell cycle distribution, and stem cell activity in each group to elucidate the possible reasons why 3D cultured cells acquire greater radioresistance.

Reduced proliferation ability in 3D cultured cells compared with 2D cultured cells has been reported in several papers [26–28], while other studies hold the opposite conclusion [29, 30]. Despite the fact that the colony formation rate in 3D cultured cells was lower than that in 2D cultured cells, the diameter of the colonies was larger, indicating a greater number of cells per colony. Reasons for the low colony formation rate and large diameters might be that 3D cultured cells migrated or gathered to form larger colonies, or there were hypoxic necrotic cells in the 3D culture cell mass. Another phenomenon observed in the present study was G1/S phase arrest occurring in 3D cultured cells. This was previously also observed in 3D cultured human adipose-derived stromal cells [26] and in tumors treated with drugs or radiation [31, 32]. A previous study demonstrated a correlation between radioresistance and the G1 phase of the cell cycle, particularly in *KRAS* mutant cells arrested in the G1 phase [33]. Several studies have shown that CSCs are responsible for tumor metastasis, ionizing radioresistance, and anti-cancer medication resistance [34, 35]. Representative CSC markers have been suggested, such as OCT4, SOX2, CD44, CD133, and CD166 [36, 37]. CD133, SOX2, OCT4, and CD44 are CSC markers for NPC [38]. Gang et al. showed that the gene and protein expression levels of OCT4, SOX2, NANOG, LIN28, and miR-302a were upregulated in three different cell lines in 3D cultures compared with 2D cultured cells [39]. We quantified the number of tumor stem cells in CNE-1 using soft agar colony assay and SOX2, CD44 protein expression levels were determined by western blot analysis. Consistent with prior research, the proportion of tumor stem cells was considerably higher in the 3D groups, indicating that tumor stem cells can better preserve their features in a 3D culture microenvironment. In conclusion, in a 3D culture environment, tumors may resist radiotherapy by G1 phase arrest, maintaining low proliferation and high stem cell activity.

The majority of our findings are in line with expectations, but some conclusions are different from the conventional cognition of 2D culture models. The wounding healing assay confirmed that the migration rates of cells in the 3D groups were significantly enhanced. In 3D cultured cells, however, the expression of epithelial-derived E-cadherin was increased while that of stromal-derived Vimentin, Slug, ZEB-1, and N-cadherin was decreased. This is contradictory to the conventional EMT tumor metastatic route [40], suggesting that the EMT pathway was not the main mechanism regulating the migration of 3D cultured tumor cells. It has been reported that ECM reorganization drives extreme changes in integrin signaling pathways fundamental for tumor development and response to radiotherapy [41, 42]. Multiple investigations on normal and malignant cells have demonstrated that adherence to the ECM increases resistance to ionizing radiation, chemotherapy, and molecular treatments. These phenomena are called cell adhesion-mediated radioresistance and cell adhesion-mediated drug resistance [16]. Studies in normal and tumor cells, including glioblastoma, pancreatic carcinoma, and breast cancer cells, documented that adhesion to the ECM enhanced resistance to ionizing radiation [43, 44]. Our results showed that the protein expression of integrin β1 in 3D cultured cells was higher than in 2D cultured cells. Members of the integrin β1 subfamily bind to FN, collagen, and laminins. The binding sites in the integrin β tail mediate interactions with many cytoskeletal components and regulate cell adhesive functions [45]. Studies have shown that the elevated expression of integrin β1 is associated with the proliferation, migration, invasion, and even the chemotherapeutic treatment susceptibility of carcinoma [46, 47]. Therefore, we believe that, unlike the increased migratory capacity commonly attributed to EMT, the difference in migratory capacity between 2D and 3D cultured cells in our study stems from the effect of cell adhesion patterns.

Co-blending was used to create collagen/alginate IPN hydrogels in this study. Collagen was maintained at a constant concentration, whereas the alginate concentration was altered. Since alginate is an inert substance with no cell-binding sites, a change in its concentration in the matrix will merely change the stiffness. Considering the benefits of our single modulation of on-surface 3D matrix stiffness based on alginate, we investigated if stiffness could affect radiosensitivity. The results demonstrated that CNE-1 cells grown on substrates of varying stiffness exhibited distinct radiation resistance. A heterogeneous stiffness map of different intratumor locations in tumors has been reported [48]. Tumor stiffness, which could be associated with tumor grade and survival in patients, is measured by magnetic resonance elastography (MRE) clinically [49]. Additionally, there has been research on how ECM stiffness affects tumor shape, treatment resistance, and tumor growth and invasion [48, 50–52]. The underlying mechanism may be that during tumor initiation and progression, complex structural changes in the ECM and cytoskeletal architecture are generated [53, 54]. Integrin β1 is a crucial molecule for sensing stiffness. On high-stiffness PA hydrogels, the integrin β1/FAK/ERK1/2/NF-κB signaling pathway was reported to be activated in SMMC-7721 cells [55]. Therefore, it can be inferred from prior research and our experimental findings that integrin β1 play a key role in the radioresistance associated with ECM stiffness in 3D cultured cells. However, more experiments are needed to further verify this inference.

This work indicated that ECM may regulate the malignant behaviors of NPC cells, such as tumor proliferation, cell cycle distribution, migration, adhesion ability, and stem cell proportion. Moreover, we produced a 3D tumor cell model varying the ECM stiffness and radioresistance of NPC cells was correlated with the stiffness value. Limitations also exist in our research, for example, these results were obtained in vitro and the underlying molecular mechanisms, such as whether radioresistance results from dsDNA repair or cancer cell stemness, should be further studied. Patient-derived tumor organoid model combines primary culture and clinical characteristics maybe a better research model.

## Conclusion

Tumor biological behaviors of NPC cells in 3D system was obviously different from that of 2D. Radioresistance of NPC cells increased with the stiffness of ECM decreasing, whose possible mechanism could be cells staying in the G1 phase, decreasing proliferation and Integrin β1-associated cell adhesion.

## Supplementary Information

**Additional file 1: Fig. S1.** The proliferation of CNE-1 cells on different On-Surface 3D matrix stiffness. (**A**) After successful establishment of the hydrogel adjusting stiffness culture system, the proliferation of CNE-1 cells on different stiffness substrates by colony formation assay were observed. The colony formation on different stiffness substrates under the microscope on the 1st, 3rd, 5th and 7th day (scale bar 100 μm). (**B**) Colony diameters (*P < 0.05) of CNE-1 cells on different stiffness substrates

## Data Availability

All data are available within the article and supplementary files, or from the authors upon request.
