# Supplementary material for: Extracellular matrix stiffness mediates radiosensitivity in a 3D nasopharyngeal carcinoma model
Source: Cancer Cell Int. 2022 Nov 19;22:364. doi: 10.1186/s12935-022-02787-5 (PMC9675143; doi:10.1186/s12935-022-02787-5)
Supplement: Supplementary file 1 — Additional file 1: Fig. S1. The proliferation of CNE-1 cells on different On-Surface 3D matrix stiffness. (A) After successful establishment of the hydrogel adjusting stiffness culture system, the proliferation of CNE-1 cells on different stiffness substrates by colony formation assay were observed. The colony formation on different stiffness substrates under the microscope on the 1st, 3rd, 5th and 7th day (scale bar 100 μm). (B) Colony diameters (*P < 0.05) of CNE-1 cells on different stiffness substrates [file 12935_2022_2787_MOESM1_ESM.docx]

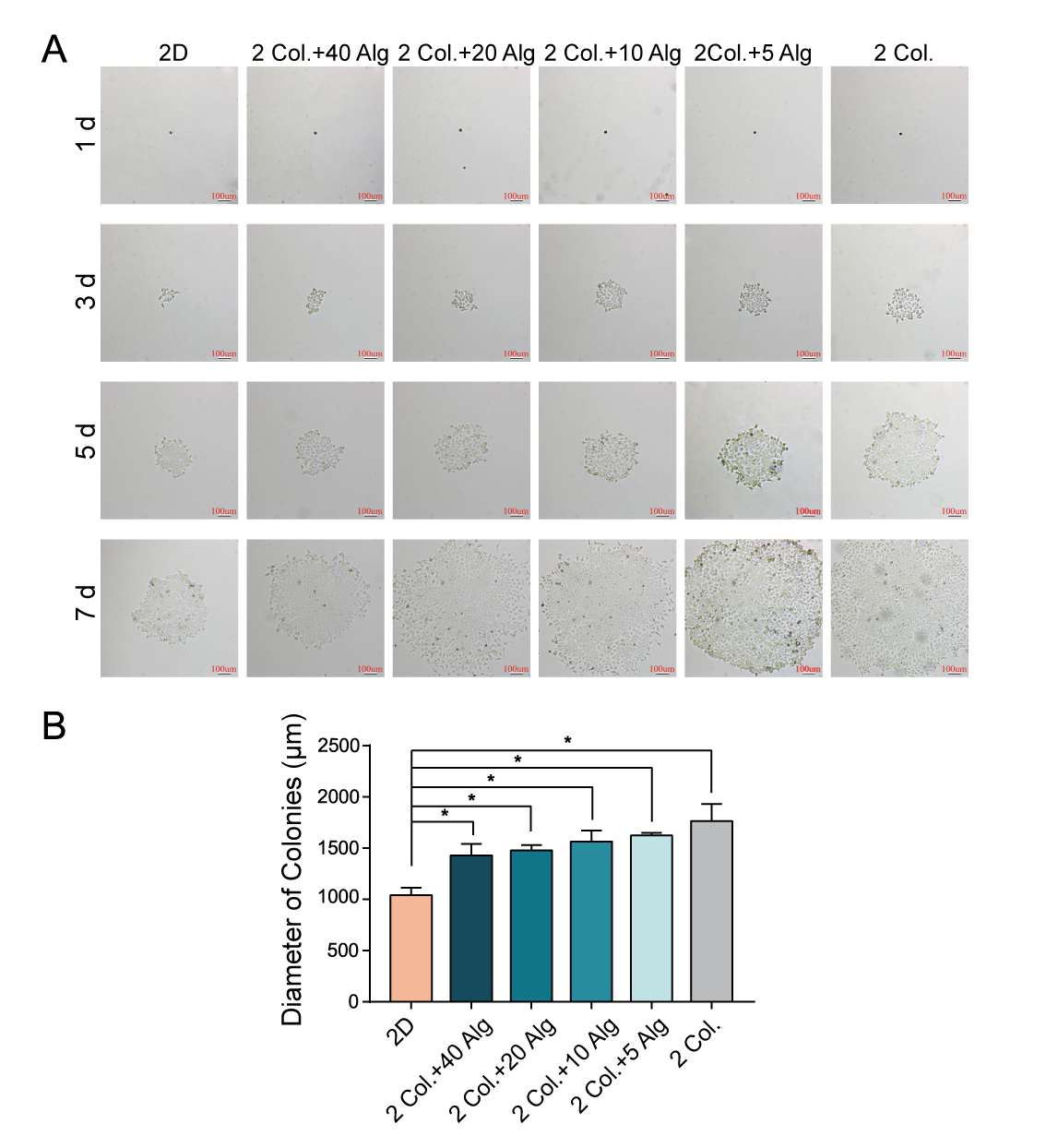


**Fig. S1** The proliferation of CNE-1 cells on different On-Surface 3D matrix stiffness. (**A**) After successful establishment of the hydrogel adjusting stiffness culture system, the proliferation of CNE-1 cells on different stiffness substrates by colony formation assay were observed. The colony formation on different stiffness substrates under the microscope on the 1st, 3rd, 5th and 7th day (scale bar 100 µm). (**B**) Colony diameters (**P*<0.05) of CNE-1 cells on different stiffness substrates.
